# A 67-year-old man with metastatic carcinoma ex pleomorphic adenoma of the parotid gland: case report and review of the literature

**DOI:** 10.1093/jscr/rjag283

**Published:** 2026-04-30

**Authors:** Angumbwike Mwakitwange, Abitalis C Mayengela, Jeremia J Pyuza, Deardiana G Sengelela, Furaha Kasyupa, Gilbert Nkya, Angela Pallangyo, Patrick Amsi, Alex Mremi

**Affiliations:** Department of Pathology, Kilimanjaro Christian Medical Center, PO Box 3010, Moshi, Tanzania; Department of Pathology, KCMC University, PO Box 2240, Moshi, Tanzania; Department of Pathology, Kilimanjaro Christian Medical Center, PO Box 3010, Moshi, Tanzania; Department of Pathology, KCMC University, PO Box 2240, Moshi, Tanzania; Department of Pathology, Kilimanjaro Christian Medical Center, PO Box 3010, Moshi, Tanzania; Department of Pathology, KCMC University, PO Box 2240, Moshi, Tanzania; Department of Pathology, Kilimanjaro Christian Medical Center, PO Box 3010, Moshi, Tanzania; Department of Pathology, KCMC University, PO Box 2240, Moshi, Tanzania; Department of Pathology, Kilimanjaro Christian Medical Center, PO Box 3010, Moshi, Tanzania; Department of Pathology, KCMC University, PO Box 2240, Moshi, Tanzania; Department of Pathology, Kilimanjaro Christian Medical Center, PO Box 3010, Moshi, Tanzania; Department of Pathology, KCMC University, PO Box 2240, Moshi, Tanzania; Department of Pathology, Kilimanjaro Christian Medical Center, PO Box 3010, Moshi, Tanzania; Department of Pathology, KCMC University, PO Box 2240, Moshi, Tanzania; Kilimanjaro Clinical Research Institute, PO Box 2236, Moshi, Tanzania; Department of Pathology, Kilimanjaro Christian Medical Center, PO Box 3010, Moshi, Tanzania; Department of Pathology, KCMC University, PO Box 2240, Moshi, Tanzania; Kilimanjaro Clinical Research Institute, PO Box 2236, Moshi, Tanzania; Department of Pathology, Kilimanjaro Christian Medical Center, PO Box 3010, Moshi, Tanzania; Department of Pathology, KCMC University, PO Box 2240, Moshi, Tanzania; Kilimanjaro Clinical Research Institute, PO Box 2236, Moshi, Tanzania

**Keywords:** carcinoma ex pleomorphic adenoma, parotid gland, facial nerve palsy, malignant salivary gland tumor, metastasis, long-term follow-up

## Abstract

Carcinoma ex pleomorphic adenoma (Ca ex PA) is an uncommon but highly aggressive malignancy arising from long-standing pleomorphic adenoma. Malignant transformation typically presents with rapid enlargement, pain, and facial nerve involvement. Metastatic spread is exceptionally rare. We describe a 67-year-old man with a decades-long right parotid mass that underwent malignant change after prior surgical excision confirmed Ca ex PA. After being lost to follow-up, he re-presented with neck stiffness, hearing loss, and facial nerve palsy. Imaging demonstrated a recurrent parotid mass infiltrating adjacent space, accompanied by cervical lymphadenopathy and bilateral pulmonary nodules suggestive of metastases. This case highlights the severe consequences of untreated or inadequately followed salivary gland neoplasms and underscores the necessity of early excision, thorough histopathologic assessment, and consistent long-term surveillance to prevent advanced Ca ex PA.

## Introduction

Pleomorphic adenoma (PA) is the most common benign tumor of the salivary glands, accounting for ~60%–70% of all parotid neoplasms [1]. Although typically indolent, long-standing or recurrent PAs may undergo malignant transformation into carcinoma ex pleomorphic adenoma (Ca ex PA), a highly aggressive entity with significant morbidity and mortality [1, 2]. According to the World Health Organization (WHO) 2022 Classification of Head and Neck Tumors, Ca ex PA is categorized by the extent of invasion: non-invasive (intracapsular), minimally invasive (≤1.5 mm extracapsular invasion), and frankly invasive (>1.5 mm extracapsular invasion) [3]. The risk of malignant transformation increases with tumor duration, advanced patient age, incomplete excision, and recurrence [4, 5].

Clinically, Ca ex PA often presents as a rapidly enlarging mass with pain, skin fixation, regional lymphadenopathy, or facial nerve dysfunction—features that strongly suggest malignant transformation [1, 2]. The most common malignant subtypes include adenocarcinoma not otherwise specified (NOS) and salivary duct carcinoma, although rarer histology such as myoepithelial carcinoma, mucoepidermoid carcinoma, and adenoid cystic carcinoma are occasionally encountered [2, 6].

We report a rare and remarkable case of Ca ex PA arising in a parotid lesion that had been present since childhood, highlighting the extreme natural history of untreated disease and emphasizing the importance of early excision and long-term follow-up to prevent malignant transformation.

## Case presentation

A 67-year-old male presented to our facility with a progressively enlarging right parotid mass and a history of swelling in the same region since the age of six. One year prior, he had undergone a total parotidectomy at another institution, during which a well-circumscribed tumor confined to the superficial lobe of the parotid gland was excised along with an enlarged lymph node. Histopathological examination at that time revealed a pleomorphic adenoma with areas of malignant transformation consistent with Ca ex PA. The patient was lost to follow-up after surgery.

He re-presented 1 year later with progressive neck stiffness, right-sided facial pain, facial numbness, hearing loss, and facial nerve palsy of 2 weeks’ duration. On examination, there was a healed post-auricular surgical scar with mild distal gaping and induration of the overlying skin, accompanied by firm swelling in the right parotid region consistent with lymphoedema. No discharge was noted.

**Figure 1:**
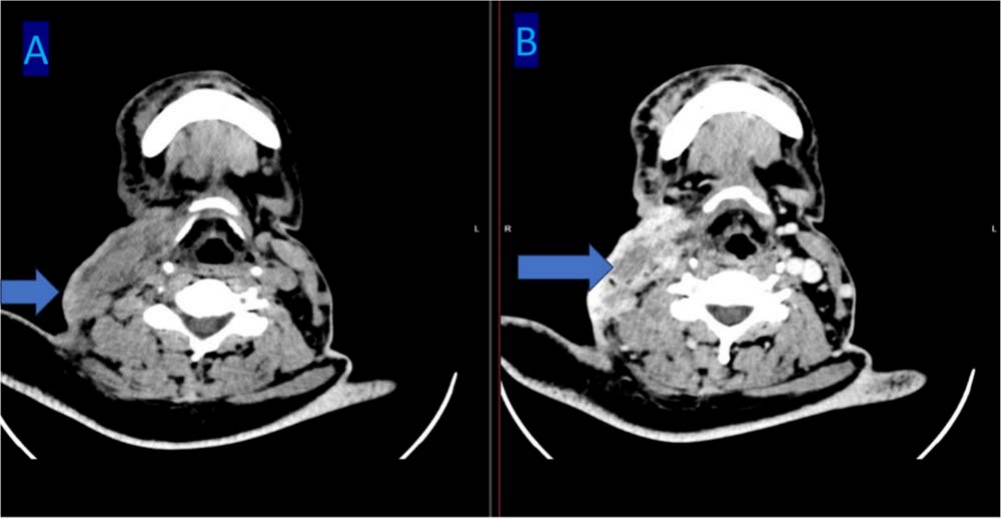
Multi slice head CT scan shows an enhancing soft tissue mass at the right parotid space, infiltrating the deep fat tissue extending between the inner border of the mandibular ramus and styloid process.

**Figure 2:**
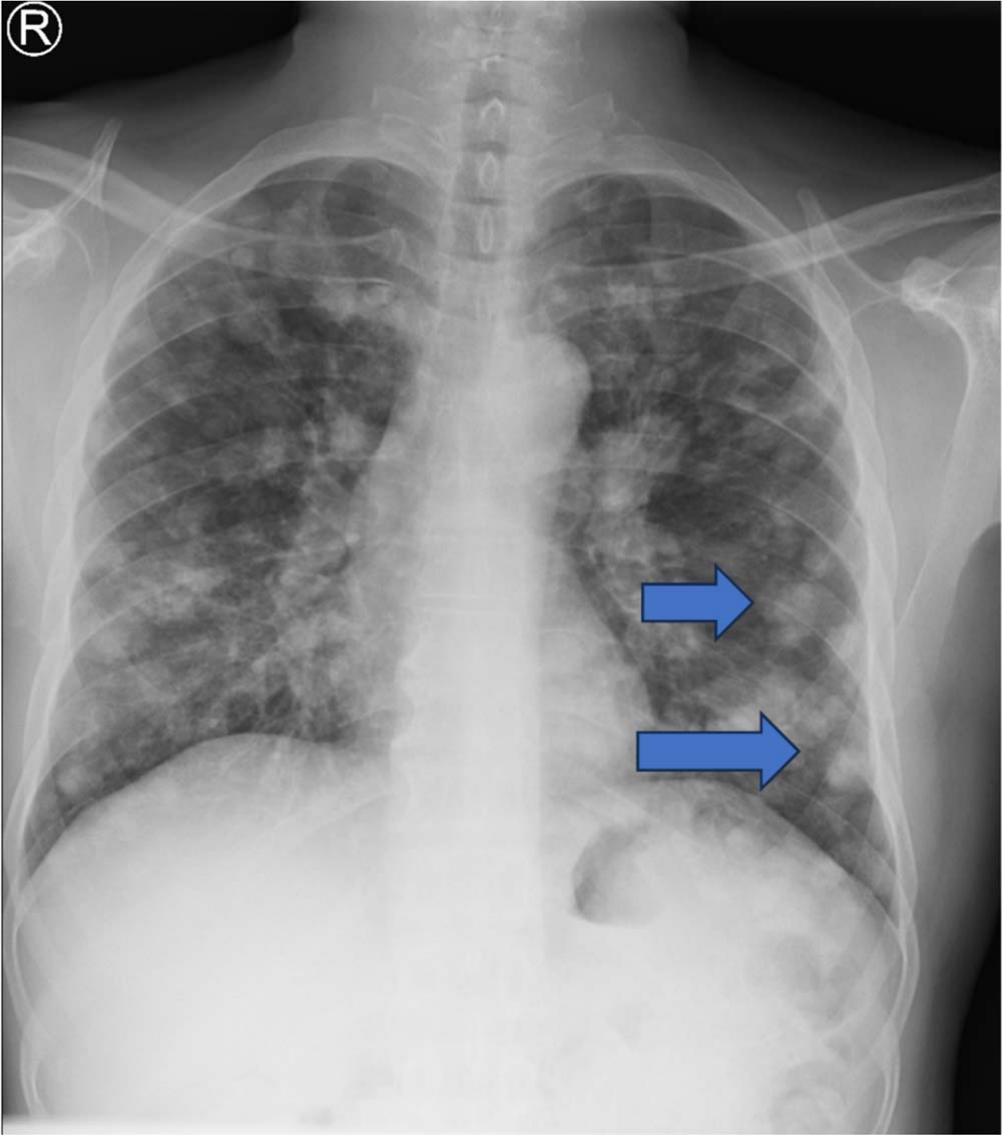
Chest X-ray (PA view) shows bilateral diffuse scattered round nodules of varying sizes suggestive of pulmonary metastases.

**Figure 3:**
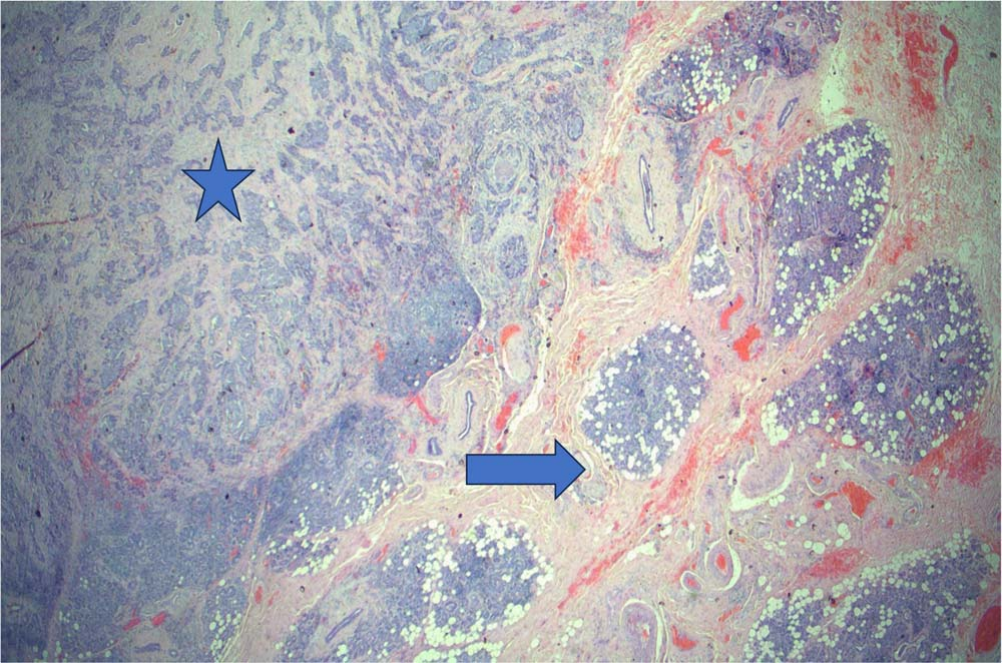
Histopathology of carcinoma ex pleomorphic adenoma showing benign (arrow) and malignant (star) components consistent with carcinoma ex pleomorphic adenoma, H&E staining at 2× original magnifications.

**Figure 4:**
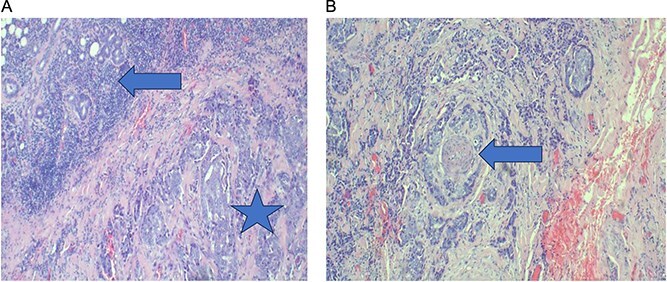
(A) Photo microscopy demonstrating interface of distinct components of carcinoma ex pleomorphic adenoma; a carcinomatous component comprised of poorly differentiated adenocarcinoma (salivary duct carcinoma) (star) and benign glandular elements (arrow). (B) Photo microscopy showing infiltrative tumour demonstrating neurotropism. H&E staining at 10× original magnifications.

Routine laboratory investigations were unremarkable. Multislice high-definition computed tomography (CT) scan of the head and neck revealed a heterogeneously enhancing soft-tissue mass occupying the right parotid space, replacing the deep fat and extending between the mandibular ramus and styloid process into the masticator and carotid spaces. There was associated ipsilateral skin thickening, near-total opacification of the right mastoid air cells, and multiple enlarged bilateral cervical and supraclavicular lymph nodes, the largest measuring 1.6 cm in short axis. Multiple bilateral pulmonary nodules were observed within the visualized lung fields (Fig. 1). Chest radiography confirmed diffuse, scattered round nodules of varying sizes, suggestive of pulmonary metastases (Fig. 2). Review of the previously excised tissue confirmed the diagnosis of invasive Ca ex PA, as reported earlier. The pathologist reported a tumor comprised of residual benign ductal and myoepithelial components within a hyalinized stroma, juxtaposed with infiltrating malignant glands composed of atypical polygonal cells showing nuclear pleomorphism, prominent nucleoli, and perineural invasion (Figs 3–5). Following multidisciplinary discussion, the patient was referred to the oncology service for further evaluation and management. Currently the patient is kept on palliative chemoradiotherapy.

## Discussion

Carcinoma ex pleomorphic adenoma (Ca ex PA) is an uncommon and aggressive malignant transformation of a pleomorphic adenoma (PA), accounting for ~3%–6% of all salivary gland malignancies and typically arising in the parotid gland. The risk of malignant transformation increases with tumor duration—estimated at 1.6% after 5 years and up to 10% after 15 years of untreated disease [1, 4, 7]. The present case demonstrates an extreme example of this phenomenon, where a benign parotid PA persisted for six decades before evolving into metastatic Ca ex PA.

Several clinical ‘red flags’ suggest malignant transformation, including rapid tumor enlargement, pain, facial nerve dysfunction, and fixation to underlying structures [2, 5]. Our patient presented with neck stiffness, pain, and right-sided facial nerve palsy—classic indicators of local invasion. Imaging findings further supported malignancy, showing an irregular, heterogeneously enhancing mass infiltrating the masticator and carotid spaces, along with multiple pulmonary metastases. Such extensive disease at diagnosis reflects the aggressive nature of invasive Ca ex PA.

Histopathologically, Ca ex PA is defined by the coexistence of residual benign PA elements and areas of frank carcinoma. The most frequent malignant subtypes are salivary duct carcinoma and adenocarcinoma not otherwise specified (NOS), though myoepithelial, mucoepidermoid, and adenoid cystic carcinoma variants have been described [2, 6]. The presence of perineural invasion, as seen in our case, is a hallmark of poor prognosis and correlates strongly with facial nerve palsy. Immunohistochemistry, although not performed here, can aid classification and prognostication—markers such as HER2, androgen receptor, p53, and Ki-67 have diagnostic and therapeutic implications [6, 7].

From a management perspective, complete surgical excision with clear margins remains the mainstay of treatment for localized disease. Adjuvant radiotherapy is often indicated for invasive tumors or those with positive margins, perineural invasion, or lymph node metastases. Malignant areas extending beyond tumor capsule, carry a poor prognosis, the 5-year survival ranging from 25% to 65%, regional lymph nodes metastases occurring in 25% of the cases [3]. Small tumor size, histological grading of the malignant component and completeness of the surgical excision are favorable outcome indicators. The single most reliable prognostic marker is the extent of tumor infiltration beyond the capsule [3]. In metastatic cases, systemic therapy (including HER2-targeted or androgen-deprivation regimens where applicable) may be considered, though prognosis remains poor with reported 5-year survival rates below 30% [1, 6].

This case also illustrates systemic challenges frequently encountered in low-resource settings—including delayed presentation, limited access to specialized oncology services, fragmented referral pathways, and financial or sociocultural barriers to care [8–12]. The patient’s six-decade history of untreated PA underscores the importance of strengthening community awareness, early surgical intervention, and structured follow-up programs. Regular surveillance every 6–12 months in the first 5 years after surgery, and annually thereafter, is recommended to detect recurrence or early malignant transformation [3].

In summary, this case exemplifies the natural history of neglected pleomorphic adenoma and reinforces the need for early diagnosis, histopathologic confirmation, and multidisciplinary long-term monitoring to prevent advanced carcinoma ex pleomorphic adenoma.

## Conclusion

This case highlights a remarkably prolonged course of pleomorphic adenoma that underwent malignant transformation into metastatic carcinoma ex pleomorphic adenoma after six decades. The patient’s presentation with pain, facial nerve palsy, and radiologic evidence of pulmonary metastases underscores the aggressive potential of this entity when diagnosis and treatment are delayed. Early complete excision of pleomorphic adenomas, routine histopathological examination of all excised lesions, and structured long-term follow-up are essential to prevent such outcomes. In resource-limited settings, public health initiatives aimed at improving cancer awareness, referral systems, and access to oncologic care are equally vital to reduce late presentations and improve survival.
